# Numerical Modeling and Investigation of Amperometric Biosensors with Perforated Membranes

**DOI:** 10.3390/s20102910

**Published:** 2020-05-21

**Authors:** Seyed Mohsen Hashem Zadeh, Mohammadhosein Heidarshenas, Mohammad Ghalambaz, Aminreza Noghrehabadi, Mohsen Saffari Pour

**Affiliations:** 1 Department of Mechanical Engineering, Shahid Chamran University, Ahvaz 61355, Iran; mohsen.hashemzadeh@gmail.com (S.M.H.Z.); heidarshenas@hotmail.com (M.H.); noghrehabadi@scu.ac.ir (A.N.); 2 Metamaterials for Mechanical, Biomechanical and Multiphysical Applications Research Group, Ton Duc Thang University, Ho Chi Minh City 758307, Vietnam; 3 Faculty of Applied Sciences, Ton Duc Thang University, Ho Chi Minh City 758307, Vietnam; 4 Department of Mechanical Engineering, Shahid Bahonar University of Kerman, Kerman 76169-14111, Iran; mohsensp@kth.se; 5 Division of Processes, KTH Royal Institute of Technology, 114 28 Stockholm, Sweden

**Keywords:** amperometric biosensor, biosensor current, finite element method, half-time response, mathematical model

## Abstract

The present paper aims to investigate the influence of perforated membrane geometry on the performance of biosensors. For this purpose, a 2-D axisymmetric model of an amperometric biosensor is analyzed. The governing equations describing the reaction-diffusion equations containing a nonlinear term related to the Michaelis–Menten kinetics of the enzymatic reaction are introduced. The partial differential governing equations, along with the boundary conditions, are first non-dimensionalized by using appropriate dimensionless variables and then solved in a non-uniform unstructured grid by employing the Galerkin Finite Element Method. To examine the impact of the hole-geometry of the perforated membrane, seven different geometries—including cylindrical, upward circular cone, downward circular cone, upward paraboloid, downward paraboloid, upward concave paraboloid, and downward concave paraboloid—are studied. Moreover, the effects of the perforation level of the perforated membrane, the filling level of the enzyme on the transient and steady-state current of the biosensor, and the half-time response are presented. The results of the simulations show that the transient and steady-state current of the biosensor are affected by the geometry dramatically. Thus, the sensitivity of the biosensor can be influenced by different hole-geometries. The minimum and maximum output current can be obtained from the cylindrical and upward concave paraboloid holes. On the other hand, the least half-time response of the biosensor can be obtained in the cylindrical geometry.

## 1. Introduction

The detection of bio-particles (like nucleic acids, proteins, bacteria, and viruses) plays a crucial role in the early diagnosis and prevention of various diseases, such as breast and prostate cancers. Nowadays, the start of diseases and their progress can be rapidly assessed using biosensors [1,2]. A quick and precise detection can lead to the administration of appropriate medicine and reduces the possibility of reaching a critical stage of the disease [3]. Therefore, the study of such biosensors has drawn the attention of the investigators to examine the effects of bioparticle detection by carrying out analytical, numerical, and experimental research.

Typically, biosensors are made up of two elements. First, the specific biological element, usually an enzyme, is responsible for detecting a sample solution. The second element that translates biorecognition is called a transducer event into an electrical signal [4]. Amperometric biosensors work by measuring electrical current. Stability is a crucial characteristic of biosensors. The operational stability of a biosensor mainly depends on the geometry of the sensor and the applied transducer. Amperometric biosensors are reliable and relatively cheap with high sensitivity, making them practical for industrial, medical, and environmental applications [5,6]; they have been a main focus of attention mostly because of the wide range of oxidase substrates they can measure, like alcohol and cholesterol [7,8,9].

Numerous studies can be found regarding the mathematical modeling of amperometric biosensors. Early works were mainly devoted to simple geometries and one-dimensional-in-space models. The mathematical models are based on time-dependent diffusion equations with a non-linear term associated with the Michaelis-Menten kinetics of the enzymatic reaction. For instance, Baronas et al. [10] studied the impact of enzyme membrane thickness on the response of an amperometric biosensor in a simple enzyme-catalyzed reaction. They employed the Finite Difference Method (FDM) for solving a set of non-linear 1-D equations and showed that the maximum current of the biosensor is a non-monotonic function of the membrane thickness. Meena and Rajendran [6] developed the 1-D mathematical modeling of amperometric and potentiometric biosensors. They used the Homotopy perturbation method to solve the non-linear differential equations and discussed the biosensor steady-state current concerning the Damköhler number (*σ*). Djaalab et al. [11] surveyed the mathematical modeling of highly sensitive enzyme biosensor kinetics. They employed the Laplace transform method and the Heaviside expansion theorem for solving the set of time-dependent reaction-diffusion equations in a one-dimensional-in-space model of the biosensor. Djaalab et al. [11] also showed that in highly sensitive biosensors, reducing the Damköhler number leads to an increase in the substrate concentration degradation. Loghambal and Rajendran [12] developed a theoretical model for the simulation of a mediated enzyme electrode in the presence of oxygen. The homotopy perturbation method was employed to solve the system of coupled, steady-state, non-linear reaction-diffusion equations. Their results showed that the thickness of the enzyme electrode caused a significant change in both the magnitude of the current response and the general behavior of the system. Croce et al. [13] surveyed the mathematical modeling of an amperometric glucose biosensor coated with layer-by-layer outer membranes and compared the diffusion profiles of various participating species and their impact on the performance of the biosensors. They utilized the FDM scheme for their numerical simulation. Croce et al. [13] compared a humic acids/ferric cations (HAs/Fe^3+^) membrane (with a high permeability to glucose) with a poly(styrene sulfonate)/poly(diallyldimethylammonium chloride) (PSS/PDDA) membrane (as a low permeable membrane to glucose) and showed that the biosensor response for the low glucose permeable membrane (PSS/PDDA) was about 30% higher than the one with a high permeability to glucose.

Extensive studies have been devoted to the mathematical modeling of the so-called sandwich-type amperometric biosensors with multiple layers. In these devices, the enzyme layer is trapped between two membranes. These membranes control the diffusion transport of the species and also diminish the potential interferences [14]. Aziz [15] developed a 1-D mathematical model to investigate the influence of membrane permeability and also selectivity on peroxide-based glucose biosensors. Their studied biosensor layout consists of three layers of selective membrane (as interferant retardant), an enzyme layer, and also the outer layer (diffusion layer). They used FDM for the numerical simulation and revealed that the selectivity of the selective membrane plays a major role in reducing interference. Romero et al. [14] investigated the mathematical modeling of sandwich-type amperometric biosensors. They studied the reaction-diffusion mechanism within the enzymatic membrane by considering oxygen as the mediator. Romero et al. [14] employed the FDM and Fortran programming language for numerical simulation and revealed that the permeability to the analyte of the external membrane and its thickness are the most influential parameters for enhancing the response time of sandwich-type biosensors.

An outer porous or perforated membrane is mainly utilized in practical biosensors for increasing their stability and also prolonging their calibration curves [16,17,18]. Schulmeister and Pfeiffer [19] developed the mathematical modeling of a biosensor in the presence of a perforated membrane. However, the geometry of the membrane was not considered in their 1-D model. The two-dimensional-in-space mathematical modeling of biosensors was proposed by Baronas et al. [20,21]. In [21], Baronas et al. investigated the influence of the presence of a cylindrical perforated membrane on the performance of the biosensor. They assumed that the perforated membrane was completely filled with enzymes and showed that the perforation level of the membrane could strongly affect the steady-state and also half-time response of the biosensor. In the other study, Baronas et al. [20] surveyed the influence of the filling level of enzymes in the perforated membrane and revealed that the impact of the filling level on the biosensor response diminished as the radius of the hole decreased.

It is worth noting that in comparison to 1-D cases, the 2-D modeling of biosensors is highly computationally demanding and may not be efficient in some situations. As such, in another study, Baronas et al. [22] evaluated the conditions in which 1-D models are accurate enough to be employed for the mathematical modeling of biosensors. They found that 1-D models cannot be used when the hole of the perforated membrane is very small; thus, in such cases, 2-D models should be utilized to obtain accurate outcomes. In addition to this, the geometry of the perforated membrane governs the mass transport by the diffusion process and thus can affect the overall performance of amperometric biosensors. Previous surveys have significantly increased knowledge about the impact of influential parameters on the performance of biosensors. However, in all of the studies to date, only cylindrical perforated membranes have been analyzed and, to the best of the authors’ knowledge, the impact of the shape of the mentioned membrane on the steady-state current and time response of biosensors has not been addressed yet.

With the above literature review, it is now clear that this study aims to numerically survey the influence of the geometry of the perforated membrane on the performance of biosensors. For this purpose, a 2-D axisymmetric amperometric biosensor with selective and perforated membranes was analyzed. Seven different geometries were designed and modeled. The governing equations were solved using the Galerkin Finite Element Method. The results were first compared and validated with four different works. Then, the influence of the geometry of the perforated membranes on the steady-state current and also the time response of the biosensor was discussed.

## 2. Structure of the Biosensor

**Figure 1:**
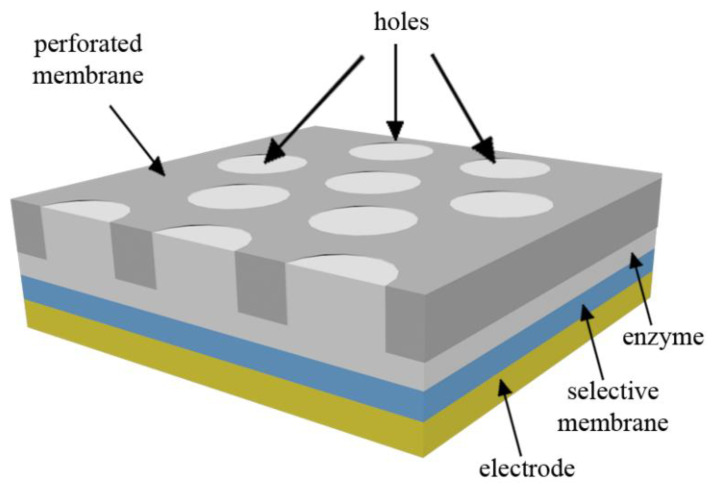
Schematic view of the biosensor with cylindrical holes.

The schematic view of the biosensor is provided in Figure 1. It is assumed that the thickness of the perforated and selective membranes is much lower than the length and width of the biosensor. The holes in the perforated membrane can be modeled to form the same unit cells with hexagonal patterns. Only half of the cross-section of the mentioned unit cell is studied because of its symmetry [20,21].

**Scheme 1:**
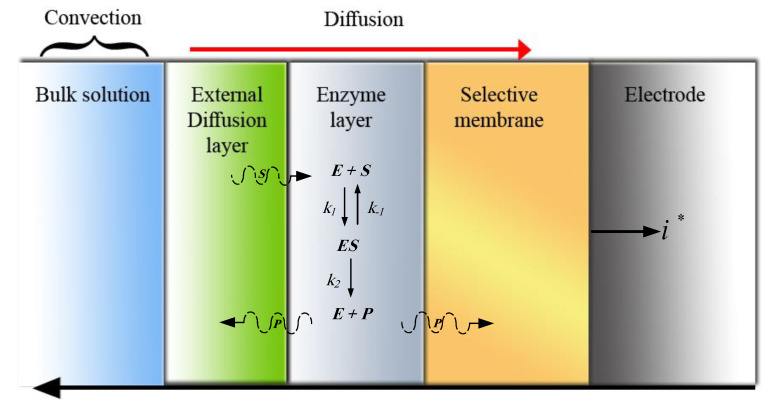
Different regions of an amperometric biosensor.

Scheme 1 illustrates various regions of the biosensor. While in the bulk solution region both convection and diffusion mechanisms (without reaction) can be found, the diffusion mode governs the other layers [14,23]. The enzyme-catalyzed reaction takes place in the enzyme region. In the selective membrane, the production detection process is performed. Finally, in the electrode, the biochemical energy from the enzyme reaction is converted by the transducer to the output current.

## 3. Formulation of the Problem

The biosensor can be mathematically modeled in a 2-D domain consisting of the discussed regions. The Michaelis–Menten model is utilized for the study of the kinetics of enzymes [24]. According to the Michaelis–Menten model, the substrate (*S*) binds to the enzyme (*E*) with the reaction rate of *k*_1_ and converts to the *ES*. The formed complex (*ES*) then dissociates during the second reaction with the reaction rate of *k*_2_ and produces the product (*P*), regenerating the enzyme [20]. It is worth noting that the rate of forward reaction rate (*k*_2_) for $ES\overset{k_{2}}{\to }E+P$ is much higher than the reverse one, and thus the reverse reaction can be neglected. Following Baronas et al. [25], the reaction model can be shown as follows:

(1) $$E+S\overset{k_{1}}{\underset{k_{-1}}{⇄}}ES\overset{k_{2}}{\to }E+P$$

Since the concentration of the substrate is much more than the *ES*, it is reasonable to consider a constant concentration for the *ES*. Thus, the concentration of the enzyme production can be calculated using the following equation [26]:

(2) $$\frac{\partial E_{S}}{\partial t}=k_{1}ES-(k_{2}+k_{-1})E_{S}$$

where *S*, *E,* and *E_S_* represent the concentrations of the substrate, enzyme, and the enzyme–substrate complex, respectively. *E*_0_ is the initial concentration of the enzyme. By replacing the expression *E* = *E*_0_ − *E_S_* in Equation (2), the production rate (*v*) can be written as follows [25,27]:

(3) $$\frac{\partial P}{\partial t}=v=k_{2}E_{S}=\frac{k_{2}E_{0}S}{\frac{k_{2}+k_{-1}}{k_{1}}+S}=\frac{V_{m}S}{K_{m}+S}$$

In Equation (3), *P* represents the concentration of the product. Moreover, the reaction rate approaches the saturated reaction rate when the *E*_0_ = *E_S_*. For this case, $V_{m}=k_{2}E_{0}$ represents the maximum reaction rate and $K_{m}=\left(k_{2}+k_{-1}\right)/k_{1}$ is the Michaelis-Menten constant, showing the concentration of the substrate when the reaction velocity is equal to one half of the maximal velocity of the reaction. The mass diffusion in the layers can be expressed over time with Fick’s law of diffusion in two or three dimensions, as follows:

(4) $$\frac{\partial c}{\partial t}=D\Delta c$$

where *t* is the time, Δ is the Laplace operator, *c* is the concentration of a component, and *D* is the corresponding diffusion coefficient. The value of the diffusion coefficient indicates the quality of the transfer, which means that the larger the diffusion coefficient, the better the diffusion mass transfer.

**Figure 2:**
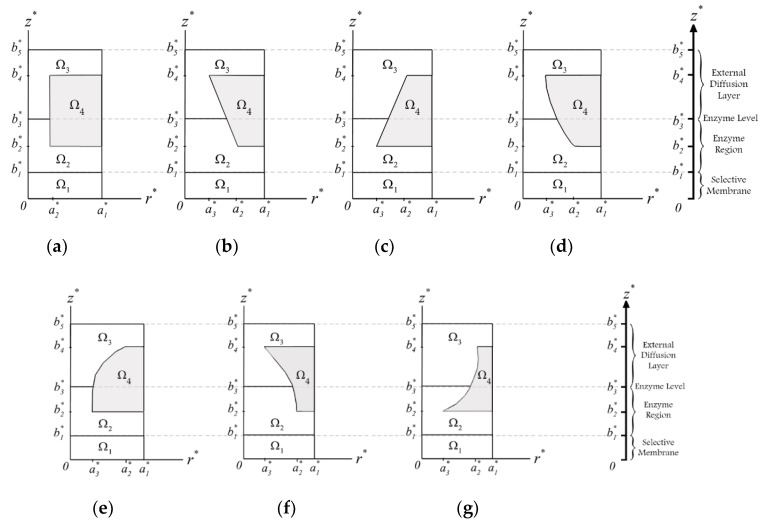
The geometries of the biosensor unit cell: (**a**) cylindrical, (**b**) upward circular cone, (**c**) downward circular cone, (**d**) upward paraboloid, (**e**) downward paraboloid, (**f**) upward concave paraboloid, and (**g**) downward concave paraboloid. Figures are not to scale.

Shown in Figure 2 are the seven studied unit cells with different geometrical designs. Except for the cylindrical one (Figure 2a), the perforated membranes were designed to be in upward or downward pairs of a circular cone, paraboloid, and concave paraboloid. As such, the minimum and maximum radii of the perforated membranes are set to be $a_{2}^{*}$ and $a_{3}^{*}$, respectively. Moreover, $a_{1}^{*}$ corresponds to the hole radius. The thickness of the selective membrane is $b_{1}^{*}$. $b_{4}^{*}-b_{2}^{*}$ and $b_{5}^{*}-b_{4}^{*}$ are the perforated membrane thickness and the diffusion layer thickness, respectively. The filling level of enzymes is shown with *b*_3_. Region Ω_1_ indicates the selective membrane, Ω_2_ specifies the layer occupied with the enzymes, Ω_3_ is the external diffusion layer, and Ω_4_ is the impermeable carrier of the perforated membrane.

It is assumed that the selected layer has a uniform thickness, and the thicknesses of both the perforated membrane and the selective membrane in the biosensor are much less than its length and width. The volume of the bulk solution is considered to be extremely high, while the substrate concentration can be assumed to be constant [28,29]. The concentration of the ES form can be assumed to be constant because the biosensor reached the steady-state condition in a short time. By using Equation (2), the maximum rate of enzymatic reaction can be obtained. Therefore, according to Figure 2, the biosensor described from the process starts (t* > 0) with the system of reaction-diffusion equations as follows:

(5a) $$\frac{\partial P_{1}^{*}}{\partial t^{*}}=D_{1}^{*}\Delta P_{1}^{*}\;\;\;\;\;\;\;\;(r^{*},z^{*})\in \Omega_{1},$$

(5b) $$\frac{\partial P_{2}^{*}}{\partial t^{*}}=D_{2}^{*}\Delta P_{2}^{*}+\frac{V_{\max}S_{2}^{*}}{K_{m}+S_{2}^{*}}\;\;\;\;(r^{*},z^{*})\in \Omega_{2},$$

(5c) $$\frac{\partial S_{2}^{*}}{\partial t^{*}}=D_{2}^{*}\Delta S_{2}^{*}-\frac{V_{\max}S_{2}^{*}}{K_{m}+S_{2}^{*}}\;\;\;\;(r^{*},z^{*})\in \Omega_{2},$$

(5d) $$\frac{\partial S_{3}^{*}}{\partial t^{*}}=D_{3}^{*}\Delta S_{3}^{*}\;\;\;\;\;\;\;\;(r^{*},z^{*})\in \Omega_{3},$$

(5e) $$\frac{\partial P_{3}^{*}}{\partial t^{*}}=D_{3}^{*}\Delta P_{3}^{*}\;\;\;\;\;\;\;\;(r^{*},z^{*})\in \Omega_{3},$$

where $P_{i}^{*}=P_{i}^{*}(r^{*},z^{*},t^{*})$ and $S_{i}^{*}=S_{i}^{*}(r^{*},z^{*},t^{*}),i=1,\;2,\;3$ are the product and substrate concentrations, respectively. The symbols r* and z* indicate the radial and axial distances, respectively, and t* denotes elapsed time. $P_{1}^{*}$ is the product concentration on the surface of the electrode; this concentration and its variation are important for measuring the change in the current of the biosensor (see Equation (26)). Here, $P_{2}^{*}$ and $P_{3}^{*}$ represent the product concentrations of the enzyme and external diffusion layers, respectively. Since the reaction product oxidizes at the electrode surface rapidly, it can be assumed that its concentration at the electrode surface $\left(z^{*}=0\right)$ is zero [21].

At the interface between the bulk solution and the biosensor $\left(z^{*}=b_{5}^{*}\right)$, the concentration of the enzyme reaction is assumed to be zero because the reaction has not yet occurred. $S_{2}^{*}$ and $S_{3}^{*}$ are the substrate concentration on the selective membrane and the perforated membrane. $D_{1}^{*}$, $D_{2}^{*}$, and $D_{3}^{*}$ represent the diffusion coefficient in these regions.

The initial conditions are described as $\left(t^{*}>0\right)$:

(6a) $$S_{2}^{*}(r^{*},z^{*},0)=0\;\;(r^{*},z^{*})\in \Omega_{2},$$

(6b) $$S_{3}^{*}(r^{*},z^{*},0)=0\;\;(r^{*},z^{*})\in {\bar{\Omega }}_{2}/\Gamma_{3},$$

(6c) $$S_{3}^{*}(r^{*},z^{*},0)=S_{0}^{*}\;\;(r^{*},z^{*})\in \Gamma_{3},$$

(6d) $$P_{i}^{*}(r^{*},z^{*},0)=0\;\;(r^{*},z^{*})\in {\bar{\Omega }}_{i}\;i=1,2,3,$$

where $S_{0}^{*}$ is the substrate concentration in the solution, ${\bar{\Omega }}_{i}$ is the area of each layer, and $\Gamma_{i}$ is the upper surface of ${\bar{\Omega }}_{i}$. Due to the oxidation of the reaction product at the electrode surface, its concentration at the electrode surface is considered to be zero ($t^{*}>0$):

(7) $$P_{1}^{*}(r^{*},0,t^{*})=0\;\;r^{*}\in [0,a_{1}^{*}].$$

Since no reaction has occurred on the boundary, $\left(z^{*}=b_{5}^{*}\right)$$\Gamma_{3}$, the concentration of the enzymatic product, has to be constant and zero. During the biosensor operation, the substrate concentration, as well as the product over the enzyme surface (bulk solution and membrane interface), remains constant; therefore ($t^{*}>0$):

(8) $$P_{3}^{*}(r^{*},b_{5}^{*},t^{*})=0\;\;r^{*}\in [0,a_{1}^{*}],$$

(9) $$S_{3}^{*}(r^{*},b_{5}^{*},t^{*})=S_{3}^{*}\;\;r^{*}\in [0,a_{1}^{*}],$$

(10) $${\frac{\partial P_{1}^{*}}{\partial r^{*}}|}_{r^{*}=0}={\frac{\partial P_{1}^{*}}{\partial r^{*}}|}_{r^{*}=a_{1}^{*}}=0.$$

The non-leakage condition for the symmetric boundaries of the unit cell is considered as follows:

(11a) $${\frac{\partial P_{2}^{*}}{\partial r^{*}}|}_{r^{*}=0}={\frac{\partial P_{2}^{*}}{\partial r^{*}}|}_{r^{*}=a_{1}^{*}}={\frac{\partial S_{2}^{*}}{\partial r^{*}}|}_{r^{*}=0}={\frac{\partial S_{2}^{*}}{\partial r^{*}}|}_{r^{*}=a_{1}^{*}}=0\;z^{*}\in [b_{1}^{*},b_{1}^{*}],$$

(11b) $${\frac{\partial P_{2}^{*}}{\partial r^{*}}|}_{r^{*}=0}={\frac{\partial S_{2}^{*}}{\partial r^{*}}|}_{r^{*}=0}=\frac{\partial P_{2}^{*}}{\partial n}=\frac{\partial S_{2}^{*}}{\partial n}=0\;\;\;\;\;z^{*}\in [b_{2}^{*},b_{3}^{*}],$$

(11c) $${\frac{\partial P_{3}^{*}}{\partial r^{*}}|}_{r^{*}=0}={\frac{\partial S_{3}^{*}}{\partial r^{*}}|}_{r^{*}=0}=\frac{\partial P_{3}^{*}}{\partial n}=\frac{\partial S_{3}^{*}}{\partial n}=0\;\;\;\;\;z^{*}\in [b_{3}^{*},b_{4}^{*}],$$

(11d) $${\frac{\partial P_{3}^{*}}{\partial r^{*}}|}_{r^{*}=0}={\frac{\partial P_{3}^{*}}{\partial r^{*}}|}_{r^{*}=a_{1}^{*}}={\frac{\partial S_{3}^{*}}{\partial r^{*}}|}_{r^{*}=0}={\frac{\partial S_{3}^{*}}{\partial r^{*}}|}_{r^{*}=a_{1}^{*}}=0\;z^{*}\in [b_{4}^{*},b_{5}^{*}],$$

(11e) $${\frac{\partial P_{2}^{*}}{\partial z^{*}}|}_{z^{*}=b_{2}^{*}}={\frac{\partial S_{2}^{*}}{\partial z^{*}}|}_{z^{*}=b_{2}^{*}}=0\;r^{*}\in [a_{2}^{*},a_{1}^{*}],$$

(11f) $${\frac{\partial P_{3}^{*}}{\partial z^{*}}|}_{z^{*}=b_{4}^{*}}={\frac{\partial S_{3}^{*}}{\partial z^{*}}|}_{z^{*}=b_{4}^{*}}=0\;r^{*}\in [a_{2}^{*},a_{1}^{*}],$$

where *n* denotes the normal vector of the hole surface. At the boundary between the two regions, it is necessary to apply matching conditions. According to the conservation of mass, the mass flux of particles at the interfaces is equal on both sides. In addition to this, it can be assumed that the substrate concentration and the enzyme product on the boundaries are equal for both sides:

(12a) $$S_{2}^{*}=S_{3}^{*}\;\mathrm{and}\;{D_{2}^{*}\frac{\partial S_{2}^{*}}{\partial z^{*}}|}_{\Gamma_{2}}={D_{3}^{*}\frac{\partial S_{3}^{*}}{\partial z^{*}}|}_{\Gamma_{3}}\;\;\;(r^{*},z^{*})\in \Gamma_{3},$$

(12b) $$P_{i}^{*}=P_{i+1}^{*}\;\mathrm{and}\;{D_{i}^{*}\frac{\partial P_{i}^{*}}{\partial z^{*}}|}_{\Gamma_{i}}={D_{i+1}^{*}\frac{\partial P_{i+1}^{*}}{\partial z^{*}}|}_{\Gamma_{i+1}}\;\;(r^{*},z^{*})\in \Gamma_{i}i=1,2.$$

In physical experiments, the anodic current generated at the electrode surface is considered as the amperometric biosensor response, which is a linear function of the reaction product gradient at the surface of the electrode. According to the Faraday and Fick laws, the current density on the electrode surface can be obtained as:

(13) $$i^{*}(t)=n_{e}FD_{1}^{*}\frac{2}{a_{1}^{*}{}^{2}}\int_{0}^{a_{1}^{*}}{\frac{\partial P_{1}^{*}}{\partial z^{*}}|}_{z^{*}=0}r^{*}dr^{*},$$

where $n_{e}$ is the number of electrons involved in a charge transfer at the electrode surface and *F* is the Faraday constant [4]. The steady-state current I* can be obtained when the concentration of the production remains constant at the sensor surface:

(14) $$I^{*}=\underset{t^{*}\to \infty }{\lim}i^{*}(t^{*}).$$

Finally, in the present study, $T_{0.5}$, half of the required time to reach the steady-state current, is used to evaluate the dynamics of the biosensor operation [25]:

(15) $$T_{0.5}=\left\{t^{*}:i^{*}(t^{*})=0.5I^{*}\right\}.$$

## 4. Dimensionless Mathematical Model

The governing Equations (6) and the corresponding boundary and initial conditions (7)–(11) are non-dimensionalized by introducing the following set of dimensionless variables:

(16) $$P_{i}=\frac{P_{i}^{*}}{K_{m}},S_{i}=\frac{S_{i}^{*}}{K_{m}},r=\frac{r^{*}}{a_{1}^{*}},z=\frac{z^{*}}{a_{1}^{*}},a_{i}=\frac{a_{i}^{*}}{a_{1}^{*}},b_{i}=\frac{b_{i}^{*}}{a_{1}^{*}},t=\frac{t^{*}D_{1}^{*}}{a_{1}^{*}{}^{2}}\;i=1,2,3.$$

Substituting Equation (16) into the governing Equation (5), the dimensionless nonlinear reaction-diffusion equations can be summarized as follows:

(17a) $$\frac{\partial P_{1}}{\partial t}=\Delta P_{1}\;\;\;\;\;\;\;\;\;(r,z)\in \Omega_{1},$$

(17b) $$\frac{\partial P_{2}}{\partial t}=D_{2}\Delta P_{2}+\sigma^{2}\frac{S_{2}}{1+S_{2}}\;\;\;\;(r,z)\in \Omega_{2},$$

(17c) $$\frac{\partial S_{2}}{\partial t}=D_{2}\Delta S_{2}-\sigma^{2}\frac{S_{2}}{1+S_{2}}\;\;\;\;(r,z)\in \Omega_{2}$$

(17d) $$\frac{\partial S_{3}}{\partial t}=D_{3}\Delta S_{3}\;\;\;\;\;\;\;\;(r,z)\in \Omega_{3},$$

(17e) $$\frac{\partial P_{3}}{\partial t}=D_{3}\Delta P_{3}\;\;\;\;\;\;\;\;(r,z)\in \Omega_{3},$$

where $D_{i}=D_{i}^{*}/D_{1}$ is the dimensionless diffusion coefficient and $\sigma^{2}=V_{\max}a_{1}^{*}{}^{2}/K_{m}D_{1}$ is the diffusion modulus (Damköhler number), which compares the rate of enzyme reaction ($V_{\max}/K_{m}$) with the mass transport through the enzyme layer ($D_{1}/a_{1}^{*}{}^{2}$) [25]. In fact, for high values of $\sigma^{2}$, the diffusion mechanism is considered to control the biosensor response, while, when it approaches zero, the enzyme kinetics prevail in the response [6,21]. The non-dimensional initial conditions are as follows:

(18a) $$S_{2}(r,z,0)=0\;\;\;\;\;\;(r,z)\in \Omega_{2},$$

(18b) $$S_{3}(r,z,0)=0\;\;\;\;\;\;(r,z)\in {\bar{\Omega }}_{2}/\Gamma_{3},$$

(18c) $$S_{3}(r,z,0)=S_{0}=S_{0}^{*}/K_{m}\;\;(r,z)\in \Gamma_{3},$$

(18d) $$P_{i}(r,z,0)=0\;\;\;\;\;\;(r,z)\in {\bar{\Omega }}_{i}i=1,2,3,$$

and the dimensionless boundary conditions read:

(19a) $$P_{1}(r,0,t)=0\;\;\;\;\;\;r\in [0,1],$$

(19b) $$P_{3}(r,b_{5},t)=0\;\;\;\;\;\;r\in [0,1],$$

(19c) $$S_{3}(r,b_{5},t)=S_{0}=S_{0}^{*}/K_{m}\;\;r\in [0,1],$$

(19d) $${\frac{\partial P_{1}}{\partial r}|}_{r=0}={\frac{\partial P_{1}}{\partial r}|}_{r=1}=0,$$

(19e) $${\frac{\partial P_{2}}{\partial r}|}_{r=0}={\frac{\partial P_{2}}{\partial r}|}_{r=1}={\frac{\partial S_{2}}{\partial r}|}_{r=0}={\frac{\partial S_{2}}{\partial r^{*}}|}_{r=1}=0\;\;z\in [b_{1},b_{2}],$$

(19f) $${\frac{\partial P_{2}}{\partial r}|}_{r=0}={\frac{\partial S_{2}}{\partial r}|}_{r=0}=\frac{\partial P_{2}}{\partial n}=\frac{\partial S_{2}}{\partial n}=0\;\;\;\;z\in [b_{2},b_{3}],$$

(19g) $${\frac{\partial P_{3}}{\partial r}|}_{r=0}={\frac{\partial S_{3}}{\partial r}|}_{r=0}=\frac{\partial P_{3}}{\partial n}=\frac{\partial S_{3}}{\partial n}=0\;\;\;\;z\in [b_{3},b_{4}],$$

(19h) $${\frac{\partial P_{3}}{\partial r}|}_{r=0}={\frac{\partial P_{3}}{\partial r}|}_{r=1}={\frac{\partial S_{3}}{\partial r}|}_{r=0}={\frac{\partial S_{3}}{\partial r}|}_{r=1}=0\;\;z\in [b_{4},b_{5}],$$

(19i) $${\frac{\partial P_{2}}{\partial z}|}_{z=b_{2}}={\frac{\partial S_{2}}{\partial z}|}_{z=b_{2}}=0\;r\in [a_{2},1],$$

(19j) $${\frac{\partial P_{3}}{\partial z}|}_{z=b_{4}}={\frac{\partial S_{3}}{\partial z}|}_{z=b_{4}}=0\;r\in [a_{2},1].$$

The non-dimensional matching conditions are:

(20a) $$S_{2}=S_{3}\;\mathrm{and}\;{D_{2}\frac{\partial S_{2}}{\partial z}|}_{\Gamma_{2}}={D_{3}\frac{\partial S_{3}}{\partial z}|}_{\Gamma_{3}}\;\;(r,z)\in \Gamma_{3},$$

(20b) $$P_{i}=P_{i+1}\;\mathrm{and}\;{D_{i}\frac{\partial P_{i}}{\partial z}|}_{\Gamma_{i}}={D_{i+1}\frac{\partial P_{i+1}}{\partial z}|}_{\Gamma_{i+1}}\;(r,z)\in \Gamma_{i}i=1,2,$$

and the non-dimensional density of the current is given by:

(21) $$i(t)=\frac{i^{*}(t)a_{1}^{*}}{2n_{e}FD_{1}^{*}K_{m}}=\int_{0}^{1}{\frac{\partial P_{1}}{\partial z}|}_{z=0}rdr,$$

and the dimensionless output current reads as:

(22) $$I=\underset{t\to \infty }{\lim}i(t).$$

To compare the effects of hole-geometry and filling level on the *i*(*t*) and *I* in different geometries, the following parameters are used [20,21,22]:

(23) $$\alpha =1-\frac{a_{2}^{*}}{a_{1}^{*}}=1-a_{2},$$

(24) $$\gamma =\frac{b_{3}^{*}-b_{2}^{*}}{b_{4}^{*}-b_{2}^{*}}=\frac{b_{3}-b_{2}}{b_{4}-b_{2}},$$

where *γ* is the level of the enzyme filling within the holes. For instance, γ=0.5 represents a case where half of the perforated membrane is filled with the enzyme. *α* represents the perforation level of the perforated membrane. For the set of studied geometrical designs, the maximum radius of the holes (*a*_3_) is considered to be constant, and thus *α* is a measure of the volume fraction of the holes in the perforated membrane. In other words, increasing the perforation level (*α*) augments the surface area of the impermeable part of the perforated membrane (Ω_4_).

## 5. Numerical Approach

The partial differential governing Equation (17) and the corresponding initial and boundary conditions (18)–(20) are solved numerically utilizing the Galerkin method. Using the basis set ${\left\{\xi_{k}\right\}}_{k=1}^{N}$, the product and the enzyme concentrations can be expanded as follows:

(25) $$P_{j}(r,z,t)\approx \sum_{k=1}^{N}P_{jk}(t)\xi_{k}(r,z)\;\mathrm{and}\;S_{j}(r,z,t)\approx \sum_{k=1}^{N}S_{jk}(t)\xi_{k}(r,z),\;j=1,2,3.$$

It should be noted that the function is the same for both variables. By substituting in Equation (17), the following residual equations can be obtained at the internal domain nodes:

(26a) $$R_{i}^{\left(1\right)}=\sum_{k=1}^{N}\frac{\partial P_{1k}}{\partial t}\int_{\Omega_{1}}\xi_{k}\xi_{i}d\Omega_{1}+\sum_{k=1}^{N}P_{1k}\left(\int_{\Omega_{1}}\frac{\partial \xi_{k}}{\partial r}\frac{\partial \xi_{i}}{\partial r}d\Omega_{1}-\int_{\Omega_{1}}\frac{1}{r}\frac{\partial \xi_{i}}{\partial r}d\Omega_{1}+\int_{\Omega_{1}}\frac{\partial \xi_{k}}{\partial z}\frac{\partial \xi_{i}}{\partial z}d\Omega_{1}\right),$$

(26b) $$\begin{array}{l} R_{i}^{\left(2\right)}=\sum_{k=1}^{N}\frac{\partial P_{2k}}{\partial t}\int_{\Omega 2}\xi_{k}\xi_{i}d\Omega_{2}+D_{2}\sum_{k=1}^{N}P_{2k}\left(\int_{\Omega_{2}}\frac{\partial \xi_{k}}{\partial r}\frac{\partial \xi_{i}}{\partial r}d\Omega_{2}-\int_{\Omega_{2}}\frac{1}{r}\frac{\partial \xi_{i}}{\partial r}d\Omega_{2}+\int_{\Omega_{2}}\frac{\partial \xi_{k}}{\partial z}\frac{\partial \xi_{i}}{\partial z}d\Omega_{2}\right)\\ -\sigma^{2}\left(\sum_{k=1}^{N}S_{2k}\int_{\Omega_{2}}\xi_{k}d\Omega_{2}\right)/\left(1+\sum_{k=1}^{N}S_{2k}\int_{\Omega_{2}}\xi_{k}d\Omega_{2}\right) \end{array}$$

(26c) $$\begin{array}{l} R_{i}^{\left(3\right)}=\sum_{k=1}^{N}\frac{\partial S_{2k}}{\partial t}\int_{\Omega 2}\xi_{k}\xi_{i}d\Omega_{2}+D_{2}\sum_{k=1}^{N}S_{2k}\left(\int_{\Omega_{2}}\frac{\partial \xi_{k}}{\partial r}\frac{\partial \xi_{i}}{\partial r}d\Omega_{2}-\int_{\Omega_{2}}\frac{1}{r}\frac{\partial \xi_{i}}{\partial r}d\Omega_{2}+\int_{\Omega_{2}}\frac{\partial \xi_{k}}{\partial z}\frac{\partial \xi_{i}}{\partial z}d\Omega_{2}\right)\\ +\sigma^{2}\left(\sum_{k=1}^{N}S_{2k}\int_{\Omega_{2}}\xi_{k}d\Omega_{2}\right)/\left(1+\sum_{k=1}^{N}S_{2k}\int_{\Omega_{2}}\xi_{k}d\Omega_{2}\right) \end{array}$$

(26d) $$R_{i}^{\left(4\right)}=\sum_{k=1}^{N}\frac{\partial P_{3k}}{\partial t}\int_{\Omega_{3}}\xi_{k}\xi_{i}d\Omega_{3}+D_{3}\sum_{k=1}^{N}P_{3k}\left(\int_{\Omega_{3}}\frac{\partial \xi_{k}}{\partial r}\frac{\partial \xi_{i}}{\partial r}d\Omega_{3}-\int_{\Omega_{3}}\frac{1}{r}\frac{\partial \xi_{i}}{\partial r}d\Omega_{3}+\int_{\Omega_{3}}\frac{\partial \xi_{k}}{\partial z}\frac{\partial \xi_{i}}{\partial z}d\Omega_{3}\right)$$

(26e) $$R_{i}^{\left(5\right)}=\sum_{k=1}^{N}\frac{\partial S_{3k}}{\partial t}\int_{\Omega_{3}}\xi_{k}\xi_{i}d\Omega_{3}+D_{3}\sum_{k=1}^{N}S_{3k}\left(\int_{\Omega_{3}}\frac{\partial \xi_{k}}{\partial r}\frac{\partial \xi_{i}}{\partial r}d\Omega_{3}-\int_{\Omega_{3}}\frac{1}{r}\frac{\partial \xi_{i}}{\partial r}d\Omega_{3}+\int_{\Omega_{3}}\frac{\partial \xi_{k}}{\partial z}\frac{\partial \xi_{i}}{\partial z}d\Omega_{3}\right)$$

In order to numerically evaluate the integral terms, the bi-quadratic function with three-point Gaussian quadrature is employed. Then, the Newton-Raphson method is used to evaluate the coefficients $P_{jk}(j=1,2,3)$ and $S_{jk}(j=1,2)$ in the nonlinear residual Equation (26). More details about the solution procedure can be found in the Reddy studies [30]. The iterative process is stopped when the convergence criterion $\sqrt{\sum {\left(R_{i}^{j}\right)}^{2}}\leq 10^{-7}\;1\leq j\leq 5$ is reached. Moreover, a time step of 0.01 s is used to evaluate the half-time response of the biosensor.

## 6. Validation of Computations

**Figure 3:**
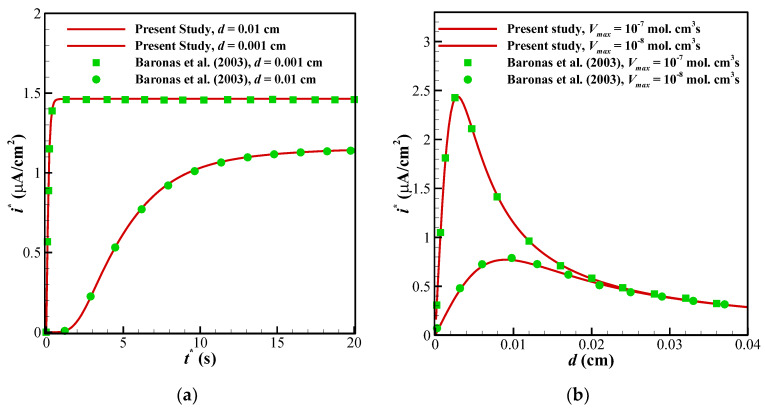
Comparison between the results of the present study with [10]: (**a**) variation in the dynamic current of the biosensor with the time and thickness of the enzyme membrane (*d*) for *V_max_* = 10^−7^ mol/cm^3^s; (**b**) dependency of the maximal current of the biosensor on the maximal enzymatic rate (*V_max_*) and the thickness of the enzyme membrane.

**Figure 4:**
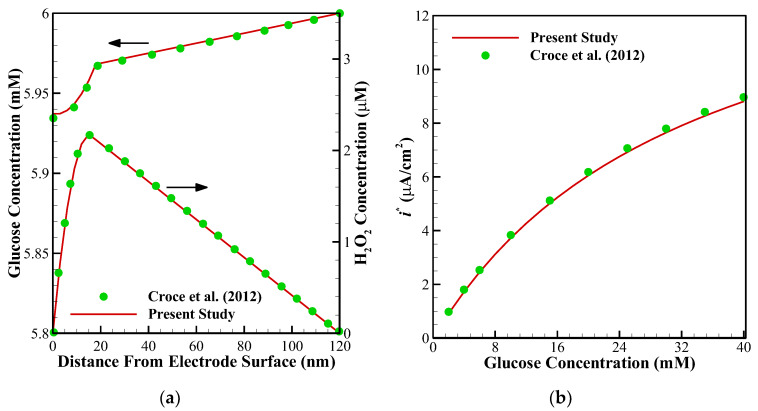
Comparison between the results of the present study with [13]: (**a**) concentration profiles of glucose and H_2_O_2_ in a multi-layer sensor system consisting of GO_x_/PPD (20 nm) and HAs/Fe^3+^ (100 nm); (**b**) dependency of the maximal current of the biosensor on the glucose concentration with 20 nm of GO_x_/PPD and 100 nm of HAs/Fe^3+^.

**Figure 5:**
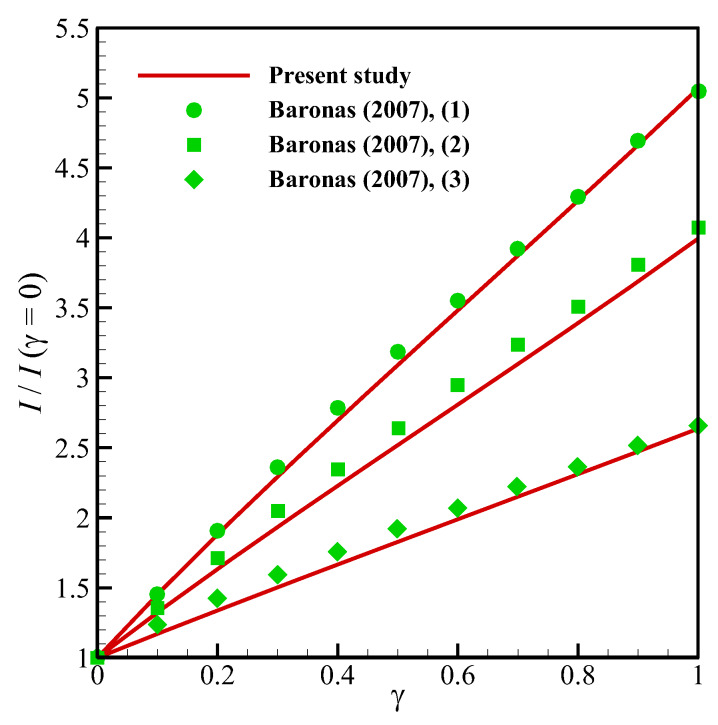
Comparison between the results of the present study and Baronas [20] in cylindrical geometry for different values of the enzyme filling level (*γ*) and the perforation level (*α*): *V_max_* = 100 μM. (1) *a*_1_ = 1 μm, *S*_0_ = 1 μM; (2) *a*_1_ = 0.8 μm, *S*_0_ = 100 μM; (3) *a*_1_ = 0.4 μm, *S*_0_ = 100 μM.

**Figure 6:**
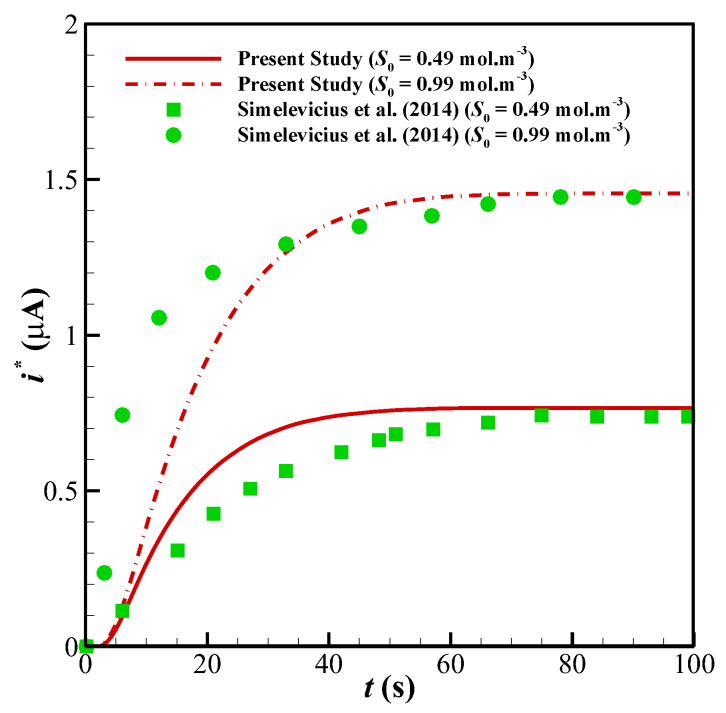
Comparison between the results of the present study with the experimental data of [31]; variation in the dynamic current of the biosensor with time and for two substrate concentrations: *S*_0_ = 0.49 and 0.99 mol/m^3^.

To ensure about the validity of the solution, the results of the present study have been compared against the different numerical and experimental results of previously published surveys. Shown in Figure 3 are the comparisons of the results of the present study with those provided by Baronas et al. [10] for a 1-D case without perforated and selective membranes (*D_s_* = *D_p_* = 3.010^−6^ cm^3^/s, *K_m_* = 10^−7^ cm^3^/s). Baronas et al. [10] employed FDM for solving the set of non-linear equations. Further, to compare the accuracy of concentration profiles, we have compared our numerical code with the concentration and current profiles of a Glucose sensor [13]. Figure 4a shows glucose and H_2_O_2_ concentration profiles in the multi-layer sensor system consisting of GO_x_/PPD and HAs/Fe^3+^ as the first and second layers. Additionally, Figure 4b, depicts the dependency of the current density of the biosensor on the concentration of the glucose. All of the simulation parameters for comparison can be found in Table 2 of [13]. In addition, to ensure the outcomes in the presence of a perforated membrane and also the perforation level (*α*) and the level of the enzyme filling (*γ*) for a cylindrical geometry (Figure 2a), the results of the present study have been validated with the work of Baronas [20] in Figure 5. Finally, to compare the presented model with an experimental study, we have developed our FEM code to take into account the influence of the mediator layer and compared our modeling with the experimental data provided by Šimelevičius et al. [31] for mediator oxidization in an amperometric glucose biosensor. The maximum value of the residual sum of squares (RSS) for Figure 3, Figure 4 and Figure 5 (comparisons with numerical approaches) are below 0.01, and for Figure 6 (comparison with the experimental data) it is around 0.35. In addition to this, the minimum value of R-square representing the similarity between the obtained results of the present study and those mentioned above is 99.8% for the numerical validations and 86.5% for the experimental comparison. This indicates an admissible agreement and very low discrepancies between the present model and all of the analyzed literature.

## 7. Results and Discussion

To study the impact of geometry on the biosensor performance, seven geometries—including cylindrical, upward circular cone, downward circular cone, upward paraboloid, downward paraboloid, upward concave paraboloid, and downward concave paraboloid—are considered. The effects of the enzyme filling level and the perforation level in these holes on the transient and steady-state current and the half-time response of the biosensor have been investigated for a range of geometrical parameters. The values of parameters are considered as $a_{1}=10a_{3}=S_{0}=1$, $D_{3}=2D_{2}=6$, $b_{5}=4b_{2}=16$, $b_{4}=7b_{1}=14$ and $\sigma^{2}=3.33\times 10^{4}$.

**Figure 7:**
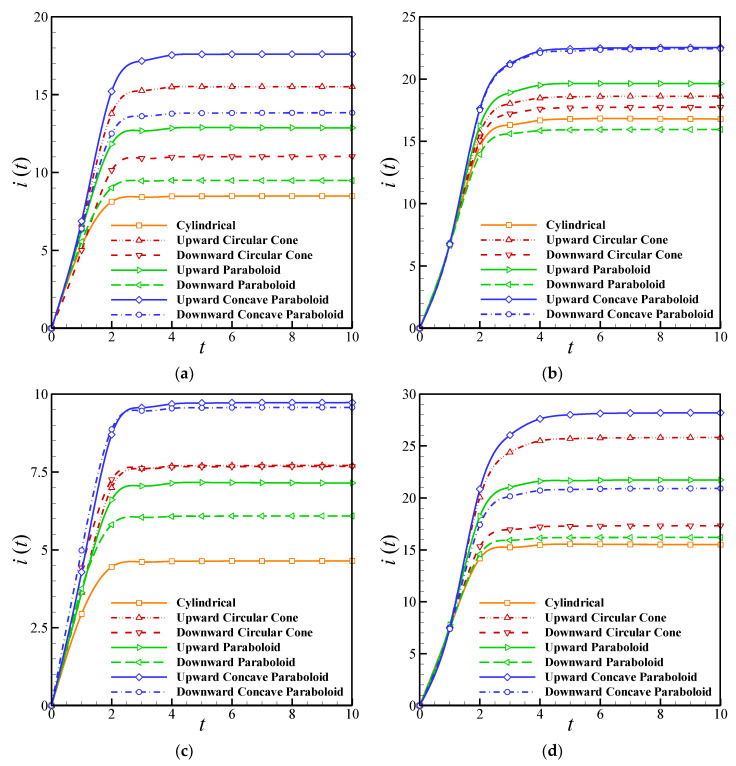
Dependency of the output current on the perforation level (γ=0.5): (**a**) α=0.2, (**b**) α=0.8. The enzyme filling level (α=0.5); (**c**) γ=0.05; (**d**) γ=0.95.

The effects of the perforation level on the transient current are shown in Figure 7a,b for various geometries. It is clear that the geometry has a significant influence on the output current of the biosensor, and the transient current is higher for the upward geometries than the downward ones. In addition to this, increasing the perforation level boosts the output current strongly. However, the difference between the current in the upward and downward geometries decreased by increasing the level of perforation for the concave paraboloid and circular cone geometries. The maximum current can be obtained from the upward concave paraboloid holes. Figure 7c,d compares the effect of the enzyme filling level on the transient current of the biosensors with different geometries. It is clear that the *γ* affects both the biosensor response time and current. The current of the biosensor for high values of *γ* is up to three times higher than that obtained when the enzyme filling level approaches zero. Moreover, the difference between the upward and downward geometries vanishes for a low level of enzyme. Finally, the minimum current is produced in the cylindrical geometry.

**Figure 8:**
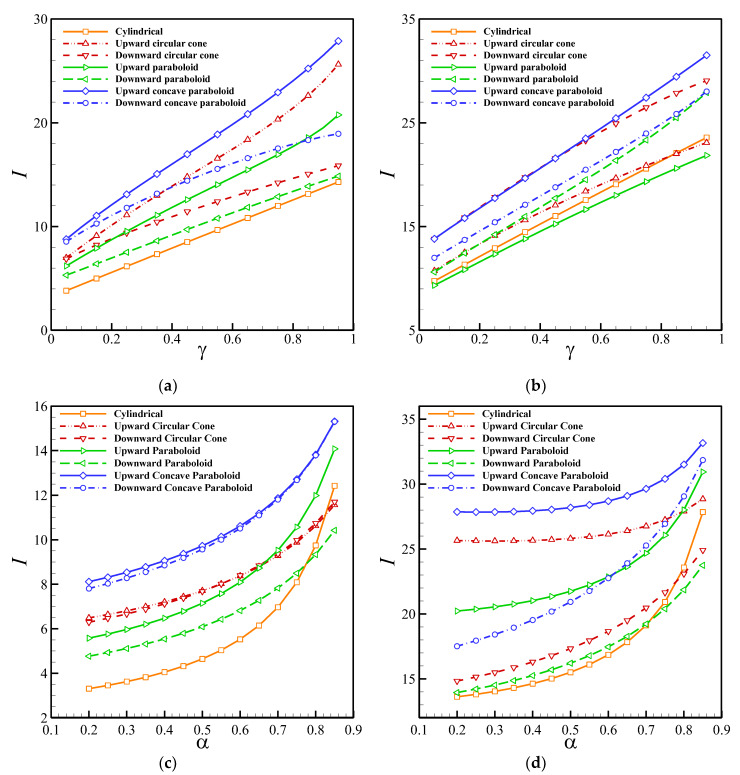
Dependency of the steady-state biosensor current on the perforation level: (**a**) γ=0.05, (**b**) γ=0.95. The enzyme filling level: (**c**) α=0.35, (**d**) α=0.8.

Figure 8 depicts the effect of the perforation level and enzyme filling level on the non-dimensional output current of the biosensor. Obviously, the current increases with the level of perforation exponentially, and the current is highly sensitive to α when it approaches 1. Moreover, increasing the perforation level reduces the difference between the maximum and minimum steady-state currents of the hole-geometries, indicating that the impact of the hole-geometry on the output current decreases as *α* increases. In contrast with *α*, the output current increases with the increment of the enzyme filling level almost linearly.

Table 1 compares the effect of the perforation level and enzyme filling level on the half-time response of the biosensor. It is evident that the cylindrical and upward concave paraboloid holes require the lowest and highest time intervals to reach the steady-state condition. In addition to this, *T*_0.5_ is a monotonous function of the enzyme filling level and perforation level, and it increases with the increment of *α* and *γ*.

## 8. Conclusions

The influence of the perforated membrane geometry on the performance of a biosensor is studied in the case of a 2-D axisymmetric model of an amperometric biosensor. For this aim, seven practical geometries—including cylindrical, upward circular cone, downward circular cone, upward paraboloid, downward paraboloid, upward concave paraboloid, and downward concave paraboloid—are taken into consideration. In addition to this, the effects of the perforation level of the perforated membrane and the filling level of the enzyme level on the transient and steady-state current of the biosensor and the half-time response are studied. The results show that the geometry of the biosensor strongly influences the sensitivity and time response of the biosensor, specifying the potential of future experimental studies. The outcomes reveal that the maximum and minimum current can be obtained from the upward concave paraboloid and circular holes, respectively. Moreover, increasing the perforation level and enzyme filling level boosts the output current strongly. On the other hand, the time response of the studied biosensor for the cylindrical geometry is lower than that for other geometries.

## Figures and Tables

**Table 1:** Influence of the *α* and *γ* on the time response (*T*_0.5_) for all of the geometries.

α	γ	Cylindrical	Upward Circular Cone	Downward Circular Cone	Upward Paraboloid	Downward Parabolic	Upward Concave Paraboloid	Downward Concave Paraboloid
0.5	0.05	0.87	1.04	0.91	0.99	0.86	1.08	0.98
0.5	0.95	1.02	1.33	1.20	1.23	1.06	1.42	1.22
0.5	0.5	0.92	1.11	1.18	1.05	0.97	1.19	1.14
0.2	0.5	0.86	1.10	0.96	1.01	0.91	1.16	1.05
0.8	0.5	1.07	1.21	1.19	1.23	1.12	1.33	1.37
